# Simple Skeletal Muscle Mass Estimation Formulas: What We Can Learn From Them

**DOI:** 10.3389/fendo.2020.00031

**Published:** 2020-02-05

**Authors:** Steven B. Heymsfield, Abishek Stanley, Angelo Pietrobelli, Moonseong Heo

**Affiliations:** ^1^Department of Metabolism-Body Composition, Pennington Biomedical Research Center, Louisiana State University System, Baton Rouge, LA, United States; ^2^Department of Surgical Sciences, Dentistry, Gynecology and Pediatrics, Verona University Medical School, Verona, Italy; ^3^Department of Public Health Sciences, Clemson University, Clemson, SC, United States

**Keywords:** sarcopenia, nutritional assessment, anthropometry, body composition, waist circumference

## Abstract

One century ago Harris and Benedict published a short report critically examining the relations between body size, body shape, age, and basal metabolic rate. At the time, basal metabolic rate was a vital measurement in diagnosing diseases such as hypothyroidism. Their conclusions and basal metabolic rate prediction formulas still resonate today. Using the Harris-Benedict approach as a template, we systematically examined the relations between body size, body shape, age, and skeletal muscle mass (SM), the main anatomic feature of sarcopenia. The sample consisted of 12,330 non-Hispanic (NH) white and NH black participants in the US National Health and Nutrition Survey who had complete weight, height, waist circumference, age, and dual-energy X-ray (DXA) absorptiometry data. A conversion formula was used to derive SM from DXA-measured appendicular lean soft tissue mass. Weight, height, waist circumference, and age alone and in combination were significantly correlated with SM (all, *p* < 0.001). Advancing analyses through the aforementioned sequence of predictor variables allowed us to establish how at the anatomic level these body size, body shape, and age measures relate to SM much in the same way the Harris-Benedict equations provide insights into the structural origins of basal heat production. Our composite series of SM prediction equations should prove useful in modeling efforts and in generating hypotheses aimed at understanding how SM relates to body size and shape across the adult lifespan.

## Introduction

One hundred years ago J. Arthur Harris and Francis G. Benedict published a short paper examining “the relationship between certain of the physical and physiological measurements of the individual” (1). Reporting their meticulous studies in 136 men and 103 women, Harris and Benedict carefully examined the relationships between body size (weight, height), body shape (surface area), and age with basal metabolic rate (BMR). Their instructive explorations led to two concluding multiple regression BMR estimation equations, one for men and the other for women that included weight, height, and age as predictor variables. Dozens of publications by others over the past century have replicated or suggested revisions to the classic Harris-Benedict equations. The ease with which the needed weight, height, and age data can be acquired balances the often-expressed concerns about their accuracy (2).

Can a similar simple approach be used to examine the relationships between body size and age with skeletal muscle mass (SM), the core element of sarcopenia (3)? Several relatively small scale studies report these instructive associations [e.g., (4–6)], but evaluations of large and diverse samples are lacking. Here, we examine the individual and combined effects of weight, height, waist circumference, and age on SM prediction in a large sample (*n* = 12,330) of National Health and Nutrition Survey (NHANES) non-Hispanic (NH) white and NH black participants (7, 8). Skeletal muscle mass was derived from dual-energy X-ray absorptiometry (DXA)-appendicular lean soft tissue as reported by Kim et al. (9). This approach requires evaluation by whole-body DXA with isolation of the extremity lean soft tissue mass during the data processing phase. Appendicular lean soft tissue mass is then calculated as the sum of upper and lower extremity lean soft tissue mass. Kim's magnetic resonance imaging-based prediction equation (9) is next used to calculate total body SM from the measured appendicular lean soft tissue mass. The NHANES waist circumference measurement method is described in the NHANES Anthropometry Procedures Manual (10). Descriptive characteristics of the sample are reported in Schuna et al. (7).

## Skeletal Muscle Mass Prediction Model

### Weight and Height

The strongest correlation between body size and SM was with weight (e.g., in representative NH white men, *R*^2^ = 0.66, *p* < 0.001), a finding consistent across the three other groups. Why is there a correlation between weight and SM? First, consider weight as having two main determinants in adults, height, and adiposity level (11). Body weight and SM both increase across adults as an approximate function of height^2^ after controlling for adiposity and age (7). People who are tall thus weigh more and have more muscle than their short counterparts. The univariate correlation between height and SM in the NH white men has an *R*^2^ of 0.32 (*p* < 0.001).

Second, greater adiposity (i.e., %fat) is also accompanied by enlargement of the SM compartment (12). People who are obese have more SM for their age and height than people who are normal weight (12). When we combine weight and height to predict SM, the *R*^2^ in NH white men increases beyond that for each component (i.e., 0.66 and 0.32) to 0.74 (*p* < 0.001). Weight and height together thus capture the independent effects of stature and adiposity on SM. As with body mass index (weight/height^2^), predicting SM from weight and height together improves our resolution of between-person body shape differences. For example, two people can weigh the same amount but differ widely in height and thus body shape.

### Age

Skeletal muscle mass decreases with age, a feature well known as part of the sarcopenia process (3). Most studies that have investigated these trends report a curvilinear (quadratic) relation between SM and age (5–8). This observation is consistent with the findings in the NH white men in whom the *R*^2^ for SM vs. age is 0.17 and increases to 0.23 (*p* < 0.001) with addition of age^2^ to the model. When age and age^2^ are added to weight and height in a multiple regression SM prediction equation (Table 1, Series 1), the *R*^2^ in the representative NH white men increases from 0.74 to 0.85 (*p* < 0.001). The improved SM predictive value with addition of age to the model arises because a young and old person who have the same weight and height will differ in their level of muscularity (5–8).

**Table 1:** Skeletal muscle mass prediction equations.

**Group**	***N***	**Equation**	**SE (*R*^**2**^)**	***P***
**Men**		**Series 1**		
NH White	4,288	SM = 0.23xW + 0.15xH – 0.058xA – 0.0005 x A^2^ – 13.2	2.3 (0.85)	<0.0001
NH Black	1,968	SM = 0.26xW + 0.16xH – 0.054xA – 0.0007xA^2^ – 14.8	2.5 (0.87)	<0.0001
Combined	6,256	SM = 0.24xW + 0.15xH – 0.071xA – 0.0004xA^2^ + 2.7xR – 14.2	2.4 (0.86)	<0.0001
**Women**				
NH White	4,108	SM = 0.19xW + 0.11xH – 0.095xA + 0.0003xA^2^ – 9.0	1.7 (0.86)	<0.0001
NH Black	1,966	SM = 0.21xW + 0.12xH – 0.132xA + 0.0006xA^2^ – 9.6	1.9 (0.87)	<0.0001
Combined	6,074	SM = 0.20xW + 0.11xH – 0.113xA + 0.0004xA^2^ + 2.0xR – 9.8	1.8 (0.88)	<0.0001
**Men**		**Series 2**		
NH White	4,288	SM = 0.46xW + 0.03xH + 0.013xA – 0.0006xA^2^ – 0.28xWC + 13.8	2.0 (0.89)	<0.0001
NH Black	1,968	SM = 0.50xW + 0.03xH + 0.031xA – 0.0008xA^2^ - 0.31xWC + 13.3	2.1 (0.91)	<0.0001
Combined	6,256	SM = 0.47xW + 0.03xH + 0.012xA – 0.001xA^2^ – 0.29xWC + 1.6xR + 13.5	2.0 (0.91)	<0.0001
**Women**				
NH White	4,108	SM = 0.24xW + 0.09xH – 0.097xA + 0.0004xA^2^ – 0.06xWC – 3.9	1.6 (0.87)	<0.0001
NH Black	1,966	SM = 0.26xW + 0.10xH – 0.120xA + 0.0006xA^2^ – 0.06xWC – 4.9	1.9 (0.88)	<0.0001
Combined	6,074	SM = 0.25xW + 0.09xH – 0.111xA + 0.0005xA^2^ – 0.06xWC + 2.0xR – 4.5	1.7 (0.89)	<0.0001

*A, age (yrs); H, height (cm); R, race/ethnicity (0, NH white; 1, NH black); SM, skeletal muscle mass (kg); W, weight (kg); WC, waist circumference (cm). Equation for predicting SM mass (kg) = 1.18xDXA appendicular lean soft tissue (kg)−0.03 x Age−0.14 (8). SM prediction equations shown in the table were developed using an adaptive lasso regression as the estimation and a k-fold validation with 100 folds. All data were analyzed using JMP Pro (JMP®, Version 14.2.0 SAS Institute Inc., Cary, NC, 1989–2019)*.

The models in the table show that even at the same weight, height, and age, NH black men, and women have more SM than their NH white counterparts. We can observe these race/ethnicity effects if we apply the models to predict SM in Reference Man and Woman defined as 25 year-old Caucasians who have the following respective weight, height, and SM: 70 kg/170 cm/28 kg and 60 kg/160 cm/17 kg (13). The race/ethnicity-specific Series 1 multiple regression models shown in the table predict that the corresponding NH white man and woman would have 26.7 and 17.6 kg of SM with larger predicted amounts in their NH black counterparts of 29.1 and 19.5 kg, a difference of about 10%. Skeletal muscle mass prediction models thus need to consider race/ethnicity as independent variables after controlling for weight, height, and age (8). These observations are consistent with the findings of Furushima et al. (14) who similarly found appendicular skeletal muscle mass prediction equations based on measures similar to those used in the current study are specific for Japanese men and women relative to those reported in non-Asian populations.

What happens to predicted SM over five decades if Reference Man and Woman's weight and height are maintained constant? The models in the table predict that NH white and NH black men would lose 5.3 and 5.7 kg of SM from age 25 to 75 years, respectively; corresponding values in women are smaller in magnitude, 3.3 and 3.5 kg. On a relative basis, the predicted percentage losses of SM across all four groups are similar (19.7 and 16.0%; 17.6 and 17.6%) and are within the commonly reported SM loss range of 3–5% per decade or 15–25% over five decades from age 25–75 years (5, 6). There is an important proviso, however, in interpreting these predictions. In reality weight and/or height when examined in cross-sectional population samples may not remain stable across the adult lifespan with one or both showing reductions in older age [e.g., (5)]. Trends in SM changes with aging thus need to be examined in relation to corresponding changes in weight and height, even in the context of longitudinal studies.

### Waist Circumference

Does adding another body size measure in the form of easily obtained waist circumference further refine our estimates of body shape and contribute to the prediction of SM? Here we can theorize that a high level of muscularity will be characterized by a proportionally larger than average amount of body mass in the chest and extremities than in the lower abdomen. Skeletal muscle resides mainly in the extremities leading to the classic body-builder phenotype: bulging chest and arms with a small waist. This muscular phenotype is notably more evident in men who typically have a larger proportion of their weight as SM compared to women. These hypothetical anatomic features are supported by the regression coefficients present in a second set of SM multiple regression models shown in the table that include waist circumference as a negative predictor variable (i.e., inverse association between waist circumference and SM). Subsequently, we observe that with a smaller waist circumference there is a larger predicted SM after controlling for weight, height, and age. As a body size measure, waist circumference alone has a significant inverse correlation with SM (e.g., *R*^2^ = 0.26, *p* < 0.001) in NH white men and *R*^2^ increases from 0.85 in the earlier combined model to 0.89 (*p* < 0.001) in the Series 2 model that includes waist circumference. Model improvements (SE and *R*^2^) with addition of waist circumference are not substantial in the women. Series 1 models based on weight, height, and age alone embody a waist circumference characteristic of the evaluated sample. Obviously, waist circumference is variable even after controlling for these three predictor variables, a phenotypic feature that can be incorporated into SM prediction using the Series 2 models.

**Figure 1:**
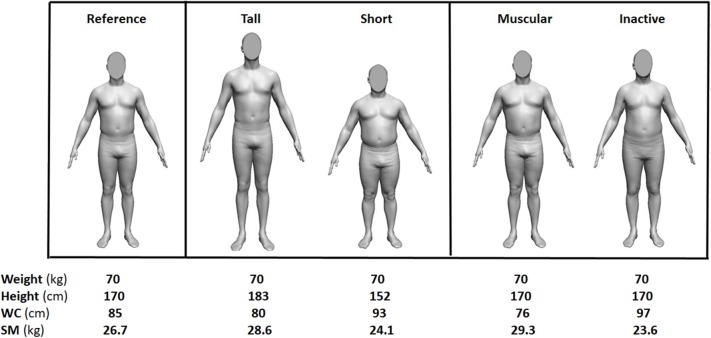
Reference Man and four phenotypic variations in height and waist circumference (WC) that relate to skeletal muscle mass (SM) differences when weight and age are held constant. The SM values were generated from the Series 2 model for NH white men, waist circumferences from a NHANES model based on weight, height, and age, and the images from a software program provided to the authors by Dr. Brian Curless at the University of Washington.

Supplemental Files are provided that incorporate series 1 and series 2 SM prediction equations in metric and US customary units along with instructions for their use. Waist circumference adds to weight, height, and age to define body shape and body composition that relate to SM. To visualize an example of these effects, a human avatar was generated with the three measures of body size (weight, height, and waist circumference) and age of Reference Man (Figure 1, left). The SM shown in the figure was calculated using the Series 2 regression model for NH white men. The middle panel of the figure shows how shape and SM vary in the Reference Man when age and weight are held constant while height is adjusted up or down. Skeletal muscle mass now varies by about 15% across the two men even though their hypothetical body mass is identical. By contrast, the panel on the right shows how varying waist circumference influences SM even when age, weight, and height are held constant. The modified Reference Man with a small waist has a larger chest and about 20% more SM than his large waist counterpart. Five different example male phenotypes identical in age and weight but varying in muscularity are thus identified through shape differences brought about by also considering two body size measurements, height and waist circumference, in the analysis.

An important feature of the Series 2 models is that people who deviate from “average” waist circumference are predicted to have less SM than their small waist counterparts of the same weight, height, and age. Schrager et al. (15) reported that after controlling for body mass index and age, people with central obesity as defined by a large waist circumference, have a pro-inflammatory state and related low handgrip strength. Based on the observations of Schrager et al. (15) we can surmise from findings of the current study that people with a relatively large waist circumference not only have less skeletal muscle mass, but an adverse metabolic state accompanied by low strength.

At present waist circumference is widely used in conjunction with weight and height, including calculated body mass index, when evaluating the health risks of excess adiposity. Our developed approach envisions use of anthropometric body dimensions and related prediction models beyond overweight and obesity to other chronic diseases, including sarcopenia, frailty, and cachexia. Additional or other circumferences might prove useful in this context, as for example in the studies of total or appendicular skeletal muscle mass prediction from three (16), two (17), and one non-waist circumference (18) models.

## Study Limitations

As with all empirical prediction models, a few limitations can be considered in ours. First, we developed our SM prediction models on a cross-sectional sample including two race/ethnic groups. Applicability in longitudinal estimations and in other race/ethnic groups is uncertain and ideally should be evaluated in future studies. Our evaluation sample also reflects adults in the general population and did not specifically include extremes such as highly trained athletes or patients with catabolic illnesses. Our focus was on SM and we did not explore other body compartments such as total body adipose tissue mass and its subcutaneous and visceral components. Skeletal muscle mass was estimated by DXA and ideally reference methods such as magnetic resonance imaging should be used in comparable future studies.

Lastly, our intent was to develop prediction models that informed on how body size and shape relate to SM across the adult lifespan. Our analyses show that three easily acquired measurements—weight, height, and waist circumference—along with age, can account for all but about 10–15% of the between individual differences in adult muscularity. Guided by the current observations, developing comparable SM prediction formulas for clinical or survey use will require careful consideration of the selected waist circumference measurement site, DXA measurement system and software, and/or magnetic resonance imaging scanner and protocol, choices that all can impact on SM estimates and developed models.

## Conclusions

By applying the Harris-Benedict strategy we thus gain new insights into the anatomic foundation underlying simple SM prediction equations based on easily acquired body size measurements and age. The potential exists to further develop these models and our understanding of anthropometric dimensions relating to SM using increasingly available three-dimensional optical scanners that can rapidly gather hundreds of body surface dimensions (19) combined with analytic strategies such as artificial intelligence/machine learning. The current study provides a roadmap for these future investigations that can improve our understanding of SM biology in relation to the development and management of sarcopenia.

## Data Availability Statement

Publicly available datasets were analyzed in this study. This data can be found here: https://wwwn.cdc.gov/nchs/nhanes/Default.aspx.

## Ethics Statement

The studies involving human participants were reviewed and approved by US Center for Disease Control. The patients/participants provided their written informed consent to participate in this study.

## Author Contributions

SH and AS designed research. SH, AS, MH, and AP conducted the research, analyzed the data, wrote the paper, and had primary responsibility for the final content.

### Conflict of Interest

SH and AP are members of the Tanita Medical Advisory Board. The remaining authors declare that the research was conducted in the absence of any commercial or financial relationships that could be construed as a potential conflict of interest.

## Abbreviations

A: age
BMI: body mass index
DXA: dual-energy x-ray absorptiometry
H: height
NH: non-Hispanic
NHANES: National Health and Nutrition Survey
R: race
W: weight/ethnicity
WC: waist circumference.
