# Supplementary material for: Simple Skeletal Muscle Mass Estimation Formulas: What We Can Learn From Them
Source: Front Endocrinol (Lausanne). 2020 Feb 5;11:31. doi: 10.3389/fendo.2020.00031 (PMC7012897; doi:10.3389/fendo.2020.00031)

**INSTRUCTIONS**

- There are two Excel files, one for metric units and the other in US customary units.
- Open the appropriate file to the "**Enter**" worksheet.
- Enter weight, height and age using appropriate units in the three yellow highlighted cells. These entries will be used to calculate skeletal muscle mass in the appropriate units and as a % of body weight in the table below for men and women based on "Model 1". There are two race/ethnicity designations (non-Hispanic <NH> white and NH black).
- If you have waist circumference as measured according to the NHANES protocol (below) you can also enter it into the appropriate yellow cell. The calculations that then follow will be based on Model 2. These models include waist circumference as an SM predictor and account for more of the individual variation in muscularity.
- Body mass index, calculated from your weight and height, is given in the Enter worksheet.

**Waist Circumference Measurement**

An absolute requirement is that measurements are made by well-trained health care workers using a standardized flexible tape at predefined anatomic locations. See the links below for measurement details.

https://www.cdc.gov/nchs/data/nhanes/nhanes_07_08/manual_an.pdf.

https://wwwn.cdc.gov/nchs/nhanes/nhanes3/AnthropometricVideos.aspx

Participants are clothed in an examination gown during these procedures. A retractable steel measuring tape can be used to take the circumference measurements. The specific NHANES waist circumference measurements instructions and accompanying illustration are as follows: Analyst "stands on the participant's right side. Palpate the hip area to locate the right ilium of the pelvis. With the cosmetic pencil draw a horizontal line just above the uppermost lateral border of the right ilium. Cross this mark at the mid-axillary line, which extends from the armpit down the side of the torso. Take the measurement: Extend the measuring tape around the waist. Position the tape in a horizontal plane at the level of the measurement mark. Use the wall mirror to ensure the horizontal alignment of the tape. Check that the tape sits parallel to the floor and lies snug but does not compress the skin. Always position the zero end of the tape below the section containing the measurement value. Take the measurement to the nearest 0.1 cm at the end of the subjects's normal expiration."

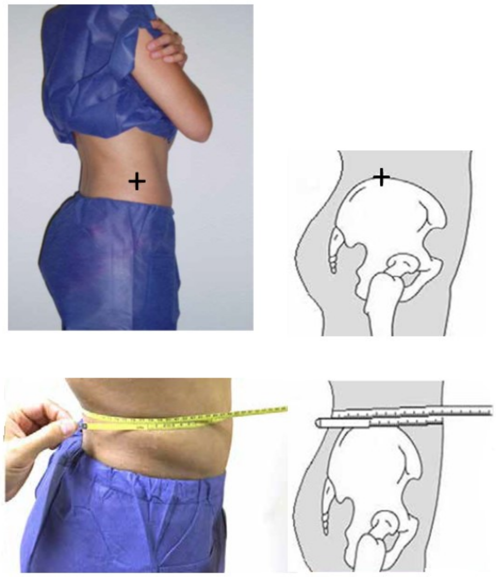

Supplement: Supplementary file 3 [file Data_Sheet_1.pdf]
